# Molecular essence and endocrine responsiveness of estrogen receptor-negative, progesterone receptor-positive, and HER2-negative breast cancer

**DOI:** 10.1186/s12916-015-0496-z

**Published:** 2015-10-05

**Authors:** Ke-Da Yu, Yi-Zhou Jiang, Shuang Hao, Zhi-Ming Shao

**Affiliations:** Department of Breast Surgery, Cancer Center and Cancer Institute, Shanghai Medical College, Fudan University, 399 Ling-Ling Road, Shanghai, 200032 P. R. China

**Keywords:** Basal-like, Breast cancer, ER−/PgR+, Endocrine responsiveness, Molecular subtype

## Abstract

**Background:**

The clinical significance of progesterone receptor (PgR) expression in estrogen receptor-negative (ER–) breast cancer is controversial. Herein, we systemically investigate the clinicopathologic features, molecular essence, and endocrine responsiveness of ER−/PgR+/HER2− phenotype.

**Methods:**

Four study cohorts were included. The first and second cohorts were from the Surveillance, Epidemiology, and End Results database (n = 67,932) and Fudan University Shanghai Cancer Center (n = 2,338), respectively, for clinicopathologic and survival analysis. The third and fourth cohorts were from two independent publicly available microarray datasets including 837 operable cases and 483 cases undergoing neoadjuvant chemotherapy, respectively, for clinicopathologic and gene-expression analysis. Characterized genes defining subgroups within the ER–/PgR+/HER2– phenotype were determined and further validated.

**Results:**

Clinicopathologic features and survival outcomes of the ER–/PgR+ phenotype fell in between the ER+/PgR+ and ER−/PgR− phenotypes, but were more similar to ER−/PgR−. Among the ER−/PgR+ phenotype, 30 % (95 % confidence interval [CI] 17–42 %, pooled by a fixed-effects method) were luminal-like and 59 % (95 % CI 45–72 %, pooled by a fixed-effects method) were basal-like. We further refined the characterized genes for subtypes within the ER−/PgR+ phenotype and developed an immunohistochemistry-based method that could determine the molecular essence of ER−/PgR+ using three markers, TFF1, CK5, and EGFR. Either PAM50-defined or immunohistochemistry-defined basal-like ER−/PgR+ cases have a lower endocrine therapy sensitivity score compared with luminal-like ER−/PgR+ cases (*P* <0.0001 by Mann-Whitney test for each study set and *P* <0.0001 for pooled standardized mean difference in meta-analysis). Immunohistochemistry-defined basal-like ER−/PgR+ cases might not benefit from adjuvant endocrine therapy (log-rank *P* = 0.61 for sufficient versus insufficient endocrine therapy).

**Conclusions:**

The majority of ER−/PgR+/HER2– phenotype breast cancers are basal-like and associated with a lower endocrine therapy sensitivity score. Additional studies are needed to validate these findings.

**Electronic supplementary material:**

The online version of this article (doi:10.1186/s12916-015-0496-z) contains supplementary material, which is available to authorized users.

## Background

The progesterone receptor (PgR) is a downstream relative of the estrogen receptor (ER), which activates the expression of PgR via the estrogen-responsive element located in the promoter region of the PgR gene. Adequate expression of PgR indicates a functional ER-α and ER-α pathway [1]. Loss of PgR expression in ER-positive (ER+) breast cancer potentially defines a subgroup with impaired function in the ER pathway, which probably gains limited benefit from endocrine therapy [2–4].

Clinically, it is generally agreed upon that all newly-diagnosed primary breast cancers should be evaluated for ER and PgR protein expression by immunohistochemistry (IHC). Although some researchers have suggested that the ER-negative/PgR-positive (ER−/PgR+) phenotype does not actually exist and may represent technical artifacts [5–7], an increasing body of evidence has shown that ER−/PgR+ tumors exist both biologically and clinically [8, 9]. Moreover, an ER−/PgR+ breast cancer cell line had been described earlier [10], indicating a mechanism of PgR expression regulation independent from ER-α.

The recently updated St. Gallen consensus on early-stage breast cancer recommends making clinical treatment decisions based on the surrogates of molecular subtypes (luminal-A, luminal-B, HER2-positive, and basal-like) defined by ER, PgR, HER2, and Ki67 [11]. The St. Gallen panelists failed to categorize the ER−/PgR+/HER2− phenotype into the four molecular subtypes, while some other guidelines treated the ER−/PgR+/HER2− phenotype as a luminal-B subtype. The ER−/PgR+ group accounts for 1 − 5 % of all breast cancers [2, 8]. Even after repeated reassessment of ER and PgR in these cases as the American Society of Clinical Oncology/College of American Pathologists (ASCO/CAP) guidelines recommend [12], at least 50 % of ER−/PgR+ remained [2, 5].

Some efforts have been made to reveal the molecular essence of ER−/PgR+ breast cancer. Using gene-expression profile information, Itoh et al. [13] proposed that, among these patients, 20 % were luminal-like and 65 % were basal-like, indicating for the first time that ER−/PgR+ breast cancer is a mixed group. In the current study, we included four large cohorts of breast cancer cases and systemically studied the clinical features and molecular essence of the ER−/PgR+ phenotype. Furthermore, we established a feasible and reliable IHC-based method to determine the subtype of each ER−/PgR+ case to guide individualized treatment. Because HER2+ breast cancers represent a biologically distinct subgroup [14], we excluded HER2+ cases from this study.

## Methods

### Four study cohorts

Cohort 1 was obtained from the database of the Surveillance, Epidemiology, and End Results (SEER) program in the United States. Cohort 2 was retrieved from the Fudan University Shanghai Cancer Center (FDUSCC). Cohort 3 was a publicly available gene expression microarray dataset previously published elsewhere [15]. Cohort 4 was also a publicly available dataset including patients undergoing neoadjuvant chemotherapy [16]. The basic characteristics of the four cohorts are shown in Table 1. The study flowchart diagram is shown in Additional file 1: Figure S1. In addition, we analyzed 64 consecutive cases with the ER−/PgR+/HER2− phenotype from FDUSCC between 2005 and 2011 to validate IHC-based markers of subtype classification (characteristics of the 64 cases are available in Additional file 2: Table S1). The datasets (cohorts 1, 3, and 4) we used in this study are publically available and no permissions were required. The research protocols of cohorts 1, 3, and 4 were determined to be qualified for institutional review board exemption by the Ethical Committee of the Shanghai Cancer Center of Fudan University. The research protocols for cohort 2 and 64 consecutive ER−/PgR+/HER2− cases were reviewed and approved by the Ethical Committee of the Shanghai Cancer Center of Fudan University. All participants provided written informed consents.

**Table 1:** Clinicopathologic characteristics of patients with HER2-negative breast cancer included for analysis

Characteristics	Cohort 1: SEER		Cohort 2: FDUSCC		Cohort 3: Publicly available cases		Cohort 4: Publicly available NCT cases	
	N = 67,932	%	N = 2,338	%	N = 837	%	N = 483	%
Age, years (IQR)	61 (51–70)		53 (45–60)		55 (45–65)		50 (42–58)	
Tumor size								
T0–1	42,281	62.2	1,132	48.4	155	38.8	33	6.8
T2	20,253	29.8	1,004	42.9	210	52.6	244	50.5
T3–4	5,398	7.9	202	8.6	34	8.5	206	42.7
Lymph nodes								
Negative	47,078	69.3	1,168	50.2	285	47.3	150	31.1
Positive	20,854	30.7	1,161	49.8	317	52.7	333	68.9
Grade								
I	17,172	26.2	39	1.7	102	13.2	31	6.8
II	29,359	44.7	1,610	68.9	244	31.6	173	38.1
III and UD	19,129	29.1	689	29.5	426	55.2	250	55.1
Subgroup								
ER+/PgR+	50,679	74.6	1,686	72.1	391	46.7	216	44.7
ER+/PgR–	7,075	10.4	177	7.6	130	15.5	72	14.9
ER–/PgR+	561	0.8	34	1.5	36	4.3	17	3.5
ER–/PgR–	9,617	14.2	441	18.9	280	33.5	178	36.9
Median follow-up, months (IQR)	11 (5–17)		37 (25–50)		49 (20–72)		36 (21–49)	

*FDUSCC* Fudan University Shanghai Cancer Center, *IQR* Interquartile range, *NCT* Neoadjuvant chemotherapy, *SEER* Surveillance, Epidemiology and End Results program, *UD* Undifferentiated

For cohort 1, obtained from the SEER database consisting of 18 population-based cancer registries, we selected patients diagnosed with invasive breast cancer between January 1, 2010, and December 31, 2013 (SEER provides HER2 status after 2010). We identified 67,932 HER2-negative patients according to the following criteria: female, surgical treatment (either mastectomy or breast-conservation), AJCC stages I–III, pathologically confirmed invasive ductal carcinoma, unilateral, known ER/PgR/HER2 status, known time of diagnosis, and breast cancer as the first cancer at diagnosis. SEER database does not conduct central review for ER/PgR/HER2. Since we enrolled the cases after 2010, the positivity of ER and PR expression should be according to the ASCO/CAP guideline (≥1 % of tumor cells with nuclear staining) [12]. Data extraction was performed by SEER*Stat software v8.1.5 [17]. The outcome of interest was breast cancer-specific survival (BCSS), which was calculated from the date of diagnosis to the date of breast cancer death. Patients who died of other causes were censored at the date of death.

For cohort 2 from FDUSCC, we included 2,338 consecutive HER2– cases of primary operable invasive breast cancer between January 1, 2008, and December 31, 2011. This is a well-characterized series of patients, whose clinicopathologic and follow-up information were maintained on a prospective basis [18]. Patient treatments were based on St. Gallen consensus [11, 19]. The cut-off for ER/PgR positivity was ≥1 % of tumor cells with nuclear staining [12]. Pathologic HER2 status was defined according to ASCO/CAP guidelines [20]. Re-assessment of ER−/PgR+ cases was carried out routinely. The outcome for this cohort was relapse-free survival (RFS), which was calculated from the date of diagnosis to the date of the first event of local, regional, or distant metastasis of breast cancer.

For cohort 3, retrieved from 36 publicly available breast cancer microarray datasets [15], among the original 5,715 unique breast cancer with expression profiles, 837 cases were identified to be HER2-negative and had information on immunohistochemical ER, PgR, and HER2 status. The normalization of gene expression data was performed by Haibe-Kains et al. [15]. Hybridization probes were mapped to Entrez GeneID as described by Shi et al. [21]. When multiple probes mapped to the same GeneID, the one with the highest variance was used. All untreated patients had surgery, although information was not available for all datasets. The PAM50 classifier was applied to the data to determine the intrinsic subtype of each case as previously described [22]. The survival outcome of interest was RFS.

For cohort 4, we selected 483 HER2– patients who participated in a prospective Institutional Review Board-approved biomarker discovery study at MD Anderson Cancer Center as published previously elsewhere [16]. The cut-off for ER/PgR positivity was ≥1 % of tumor cells with nuclear staining. All patients received neoadjuvant chemotherapy containing a taxane/anthracycline-based regimen (followed by endocrine therapy if ER+). In our analysis, cases with indeterminate ER and PgR had been excluded, and the outcome for analysis was distant RFS (DRFS). Detailed methods for RNA purification and microarray hybridization have been reported previously [16, 23]. Gene expression profiling with Affymetrix U133 gene chips was performed. Gene expression levels were derived from multiple oligonucleotide probes on the microarray that hybridize to different sequence sites of a gene transcript (probe sets). Gene expression data are available under Gene Expression Omnibus accession number of GSE25066. The PAM50 classifier was applied to determine the subtype of each case [22].

### Gene expression measurement

In cohorts 3 and 4, each ER−/PgR+ case was assigned an intrinsic subtype by the PAM50 classifier [22]. The original gene expression profile data were only available in cohort 4. Using these original data [16], we compared the gene expressions of interest between different subtypes of ER−/PgR+. To determine the functional ER pathway, mRNA expression of estrogen-responsive genes, TFF1 (pS2), GREB1, and PDZK1, were measured [24]. Expression of basal-associated cytokeratins (CKs) and EGFR were measured [25, 26]. Moreover, because the claudin-low subgroup is associated with a specific subtype of triple-negative breast cancer, mesenchymal stem-like [27], we also measured the expression of the epithelial-mesenchymal transition-associated gene CDH1 and claudin genes to discriminate mesenchymal stem-like from basal-like [28]. Probe sets used for measurement of mRNA expression are listed in Additional file 3: Table S2. Expression data were normalized with the MAS5 algorithm, the mean was centered to 600 and log2 was transformed as previously described [13].

An endocrine therapy sensitivity score was calculated by the average log2 transformed expression values of ER, PgR, BCL2, and SCUBE2 with following measurement: (0.8*ER + 1.2*PGR + BCL2 + SCUBE2)/4 as previously described in OncotypeDX [29]. This ER group score could predict of response to tamoxifen and a higher score indicates a higher sensitivity to endocrine therapy [30, 31]. For 64 cases (consecutive cases with the ER−/PgR+/HER2− phenotype from FDUSCC) with formalin-fixed paraffin-embedded samples, the method of RNA extraction and real-time PCR is provided in Additional file 4: Supplemental Methods. PCR primers are listed in Additional file 5: Table S3.

### Immunohistochemistry

IHC was performed in the 64 cases from FDUSCC according to the standard procedure [25]. Staining patterns were as follows: cytoplasmic and/or membranous staining for EGFR and CK5, and cytoplasmic staining for TFF1 (pS2). The cutoff value for positivity for TFF1 was 10 % [32]; CK5 and EGFR scored positive if any (weak or strong) staining was observed as previously described [25]. The antibodies used were reported in our previous study [33].

### Statistical analysis

Comparisons of patient and tumor characteristics were performed using the χ^2^ test or two-sample *t*-test. Survival curves were constructed using Kaplan–Meier method and tested by log-rank test. Multivariate adjusted hazard ratios (HRs) with 95 % confidence intervals (CIs) were calculated using the Cox proportional hazards model. The Mann–Whitney test was used to test gene expression differences. To analyze the combined results, we employed a two-step approach [34]. At first, the individual participant data from each study were analyzed separately (i.e. to obtain the results of each cohort). Then, the results were synthesized in the second step using a suitable model for meta-analysis of aggregate data. The meta-analysis was conducted in adherence to the standards of quality [35]. To pool the proportions, we used the command “metaprop_one” in Stata. According to a previous study [36], the score methods are recommended for proportion interval estimates and in our study the Wilson score confidence intervals were computed. We also assessed the heterogeneity among cohorts by using Cochran χ^2^ Q statistics and *I*^2^ statistics. If *P* values <0.05 or *I*^2^ > 25 % were obtained, we determined that there was a significant heterogeneity [35]. Use of a fixed-effects method (Inverse-variance method) or a random-effects method (DerSimonian and Laird method) was performed according to heterogeneity. When we compared survival estimates of ER−/PgR+ versus ER+/PgR+ and ER−/PgR− versus ER+/PgR+, we used multivariate meta-analysis (command “mvmeta” in Stata). Multivariate meta-analysis has been described previously [37, 38]. The method we used was restricted maximum likelihood and the variance-covariance matrix was defined as “unstructured”. Statistical analyses were performed with Stata v.14.0 and SPSS v.17. Two-sided *P* <0.05 was considered statistically significant.

## Results

### Clinicopathologic features and survival outcomes of breast cancer with ER–/PgR+/HER2− phenotype

In HER2– cases, the ER−/PgR+ phenotype accounted for 0.8–4.3 % among the four cohorts, with the pooled overall proportion of 2.5 % (95 % CI, 1.4–3.6 %, by a random-effects method), which is consistent with previous reports [2, 8, 9, 39]. Of note, in the consecutive cases from cohorts 1 and 2, the overall proportion of the ER−/PgR+ phenotype was 1.1 % (95 % CI, 0.5–1.7 %, by a random-effects method).

We compared the clinicopathologic characteristics of tumors of the ER−/PgR+ phenotype with those of the ER+/PgR+ and ER−/PgR− phenotypes (Additional file 6: Table S4). ER−/PgR+ tumors were associated with significantly younger age at onset, larger tumor size, higher positive node rate, and higher grade (all *P* <0.001) compared with ER+/PgR+ tumors in cohort 1. These differences were successfully validated in most but not all the other cohorts. For instance, difference in tumor size observed in cohort 1 failed to be validated in cohort 3. When compared with the ER−/PgR− phenotype, ER−/PgR+ tumors showed characteristics that were similar to or slightly more favorable than those of the ER−/PgR− phenotype.

**Fig. 1:**
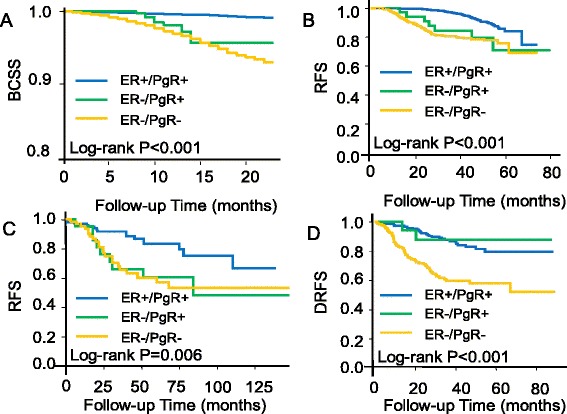
Kaplan-Meier estimates of survival are shown according to ER and PgR status in the four cohorts. (**a**) Breast cancer-specific survival (BCSS) of cohort 1; (**b**) Relapse-free survival (RFS) of cohort 2; (**c**) RFS of cohort 3; (**d**) Distant relapse-free survival (DRFS) of cohort 4. Log-rank *P* values are shown

Significant differences in survival between ER−/PgR+ and ER+/PgR+ were observed in cohorts 1–3 but not in cohort 4 either in univariate analysis (Fig. 1) or after adjustment (cohort 1: HR = 3.26 [95 % CI, 1.71–6.22], *P* <0.001 for BCSS after adjustment for age, tumor size, lymph nodes status, and grade; cohort 2: HR = 2.61 [95 % CI, 1.20–5.67], *P* = 0.016 for RFS after adjustment for age, tumor size, lymph nodes status, and grade; cohort 3: HR = 2.68 [95 % CI, 1.10–6.55], *P* = 0.030 for RFS after adjustment for age, lymph nodes status, and grade; cohort 4: HR = 1.09 [95 % CI, 0.26–4.64], *P* = 0.90 for DRFS after adjustment for age, tumor size, lymph nodes status, grade, and pathological complete response; Table 2). In contrast, there were numerical but insignificant differences between ER–/PgR+ and ER–/PgR– phenotypes. Generally, ER–/PgR+ showed survival outcomes midway between ER+/PgR+ and ER–/PgR–, although the survival curve of ER–/PgR+ was more similar to that of the ER–/PgR– cases. The fact that survival outcomes in cohorts 1–3 could not be observed in cohort 4 might be because of limited number of ER–/PgR+ cases (n = 17) and highly selected patients (with locally advanced disease and who underwent neoadjuvant chemotherapy) in that cohort. Furthermore, we investigated the pooled survival outcomes of ER–/PgR+ versus ER+/PgR+ and ER–/PgR– versus ER+/PgR+ by using multivariate meta-analysis (method: restricted maximum likelihood), the pooled HR was 2.67 (95 % CI, 1.77–4.05) for ER–/PgR+ versus ER+/PgR+ and 3.97 (95 % CI, 3.38–4.66) for ER–/PgR– versus ER+/PgR+. Taken together, the clinicopathologic features and survival outcomes of the ER–/PgR+ phenotype fell in between the ER+/PgR+ and ER–/PgR– groups but were closer to the latter.

**Table 2:** Univariate and multivariate analysis of survival for ER and PgR subgroups

Subgroups	Cohort 1: BCSS				Cohort 2: RFS				Cohort 3: RFS				Cohort 4: DRFS			
	HR (95 % CI)		P1	P2	HR (95 % CI)		P1	P2	HR (95 % CI)		P1	P2	HR (95 % CI)		P1	P2
	Univariate	Adjusted^a^			Univariate	Adjusted^a^			Univariate	Adjusted^b^			Univariate	Adjusted^c^		
ER+/PgR+	1 (ref.)	1 (ref.)	<0.001^d^		1 (ref.)	1 (ref.)	<0.001^d^		1 (ref.)	1 (ref.)	0.006^d^		1 (ref.)	1 (ref.)	<0.001^d^	
ER−/PgR+	4.67 (2.55–8.57)	3.26 (1.71–6.22)	<0.001	<0.001	2.76 (1.27–5.99)	2.61 (1.20–5.67)	0.017	0.016	2.51 (1.11–5.68)	2.68 (1.10–6.55)	0.028	0.030	0.78 (0.19–3.27)	1.09 (0.26–4.64)	0.79	0.90
ER−/PgR−	7.26 (6.10–8.64)	4.12 (3.33–5.09)	<0.001	<0.001	3.65 (2.71–4.92)	4.10 (3.03–5.54)	<0.001	<0.001	2.59 (1.36–4.93)	2.51 (1.21–5.20)	0.003	0.013	3.33 (2.16–5.15)	3.66 (2.15–6.23)	<0.001	<0.001

Here we provided both unadjusted and adjusted values of HR of BCSS/RFS/DRFS to show the survival effect of ER/PgR status

P1: Pairwise *P* value for univariate analysis (by log-rank test). ER+/PgR+ group as reference

P2: Pairwise *P* value for multivariate analysis (by Cox regression). ER+/PgR+ group as reference

*BCSS* Breast cancer-specific survival, *CI* Confidence interval, *DRFS* Distant relapse-free survival, *HR* Hazard ratio, *ref* Reference, *RFS* Relapse-free survival

^a^Adjusted for age, tumor size, lymph nodes status, and grade

^b^Adjusted for age, lymph nodes status, and grade. Tumor size is not adjusted because only half of cases have available information on it

^c^Adjusted for age, tumor size, lymph nodes status, grade, and pathological complete response

^d^Overall *P* value for univariate analysis (by log-rank test)

### Intrinsic molecular subtypes within ER–/PgR+/HER2– phenotype

Intrinsic molecular subtypes of breast cancer have been thoroughly studied [14, 40], but previous research failed to assign the ER–/PgR+ phenotype to one specific and stable molecular subtype [41]. Cohorts 3 and 4, but not cohorts 1 and 2, had available information of intrinsic molecular subtypes defined by gene expression profile. We therefore explored the distribution of known intrinsic subtypes within the ER–/PgR+ phenotype in cohorts 3 and 4. Independent cohorts 3 and 4 showed similar results and the ER–/PgR+ phenotype had a higher likelihood of being the basal-like subtype (Table 3). When we combined these two cohorts together using a meta-analytic approach (command “metaprop_one” in Stata), 30 % (95 % CI, 17–42 %, by a fixed-effects method) of ER–/PgR+ phenotype was luminal-like and 59 % (95 % CI, 45–72 %, by a fixed-effects method) were basal-like. Both the luminal-like and basal-like subtypes accounted for about 89 % of the whole ER–/PgR+ group.

**Table 3:** Relationship between immunohistochemistry-based subgroups and PAM50-based intrinsic subtypes

		Gene-expression based subtype (by PAM50 classifier)										
IHC-based subgroup (HER2–)	Total (n)	Luminal-A (n)	%	Luminal-B (n)	%	Basal (n)	%	HER2+ (n)	%	Normal-like (n)	%	*P*
Cohort 3												<0.001
ER+/PgR+	391	179	45.8	168	43.0	20	5.1	7	1.8	17	4.3	
ER−/PgR+	36	6	16.7	6	16.7	20	55.6	3	8.3	1	2.8	
ER−/PgR−	280	9	3.2	27	9.6	207	73.9	25	8.9	12	4.3	
Cohort 4												<0.001
ER+/PgR+	216	126	58.3	50	23.1	13	6.0	12	5.6	15	6.9	
ER−/PgR+	17	3	17.6	1	5.9	11	64.7	1	5.9	1	5.9	
ER−/PgR−	178	2	1.1	3	1.7	142	79.8	14	7.9	17	9.6	

*IHC* Immunohistochemistry

Because we had the original gene expression data of each case in cohort 4, we could investigate the ESR1 gene (ER) expression in the ER–/PgR+ phenotype in this cohort. A log2-transformed expression value of ≥10.18 was considered as ER+ by mRNA according to a threshold established in previous publications [13, 23]. Five of 17 (29 %; 95 % CI, 10–56 %) patients who were IHC ER– had high expression of ESR1 mRNA and may be considered as false-negative IHC results. The majority of the ER–/PgR+ phenotype (71 %) showed low ESR1 mRNA but variable PGR mRNA (Additional file 7: Figure S2), indicating the existence of a ER–/PgR+ phenotype.

### Characterized gene expression of ER–/PgR+/HER2– phenotype

**Fig. 2:**
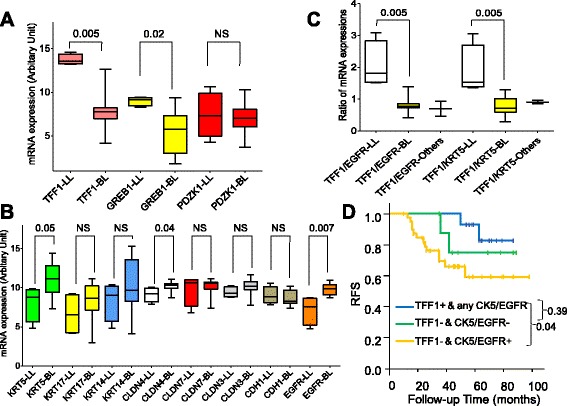
Expression of featured genes in tumors with the ER–/PgR+ phenotype. Box plots of expression of estrogen-responsive genes (**a**) and basal, claudins, and mesenchymal stem genes (**b**) for subtypes within ER–/PgR+/HER2– cases from cohort 4. (**c**) Ratio of TFF1 to EGFR or to CK5 for basal-like (n = 4), luminal-like (n = 11), and other subtypes (n = 2). *P* values are for comparisons between luminal-like and basal-like by Mann–Whitney test. The probe sets used for gene expression are 205009_at for TFF1, 205862_at for GREB1, 205380_at for PDZK1, 201820_at for KRT5, 205157_s_at for KRT17, 209351_at for KRT14, 201428_at for CLDN4, 202790_at for CLDN7, 203953_s_at for CLDN3, 201130_s_at for CDH1, and 201983_s_at for EGFR. (**d**) Kaplan–Meier estimates of relapse-free survival according to subgroups within the ER–/PgR+ phenotype using immunohistochemistry-based TFF1 (pS2), KRT5 (CK5), and EGFR. Three groups were defined as follows: luminal-like is defined as TFF1-positive and any CK5 and/or EGFR staining; basal-like is defined as TFF1-negative and positive for at least one marker of CK5 and EGFR; the remaining cases are in the undetermined group. Log-rank *P* values for pairwise comparison are shown. BL, Basal-like; LL, Luminal-like; NS, Not significant

Having found that the ER–/PgR+ phenotype was shared between luminal-like and basal-like groups at the molecular level, we further sought the characterized genes for luminal-like and basal-like ER–/PgR+ subgroups. The original gene expression data were only available in cohort 4, but not in the remaining three cohorts. Figure 2 shows the differential expression of candidate genes across different intrinsic subtypes within the ER–/PgR+ phenotype. Higher expression of TFF1 and GREB1 is significantly associated with luminal-like (Mann–Whitney test *P* = 0.005 and *P* = 0.02, respectively, Fig. 2a), while increased expression of CK5 (KRT5) or EGFR tended to be associated with basal-like (Mann–Whitney test *P* = 0.05 and *P* = 0.007, respectively, Fig. 2b). The combination of TFF1 with CK5 or EGFR significantly discriminated luminal-like ER–/PgR+ from basal-like ER–/PgR+ (Fig. 2c). Of note, the basal-like subtype within ER–/PgR+ did not show claudin-low or CDH1-low features compared with the luminal-like subtype.

### Refine the subtypes within the ER–/PgR+/HER2– phenotype by IHC markers

Based on the findings of characterized gene expression for luminal-like and basal-like ER–/PgR+, we further validated three characterized markers by IHC to determine an individual case as a certain subtype by a feasible IHC method. Because EGFR and CK5 (coded by KRT5) expression rates are not high (55–65 %) in basal-like cases [25, 42], we employed both EGFR and CK5 to single out basal-like to a large extent. We performed this analysis in the 64 ER–/PgR+/HER2– cases from our single institute between 2005 and 2011 because we could obtain their tissue samples for IHC assay but could not get formalin-fixed paraffin-embedded samples in cohorts 1, 3, and 4. We characterized the 64 cases into three groups by expression of TFF1, EGFR, and CK5. Basal-like and luminal-like subtypes were identified and constituted 63 % (40 of 64; 95 % CI, 50–74 %) and 23 % (15 of 64; 95 % CI, 14–36 %) of the tumors studied, respectively (Additional file 2: Table S1). The basal-like subgroup displayed the worst prognosis relative to the other two subgroups while the luminal-like cases tended to have the most favorable RFS (Fig. 2d). After adjustment for other prognostic factors such as age at diagnosis, tumor size, node status, and grade, the three-marker defined subgroup was an independent prognostic factor for relapse (HR of 2.4; 95 % CI, 1.17–5.03; *P* = 0.017).

### Sensitivity to endocrine therapy of subtypes within ER–/PgR+/HER2– phenotype

**Fig. 3:**
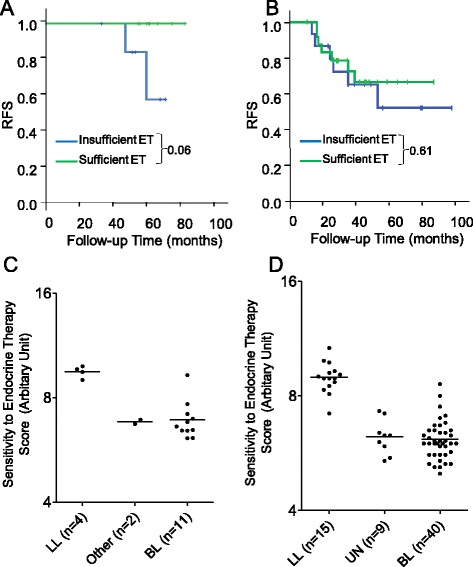
Sensitivity to endocrine therapy of subtypes within ER–/PgR+ phenotype. Kaplan–Meier estimates of RFS according to sufficient endocrine therapy or not in the luminal-like (**a**) and basal-like (**b**) subgroup in 55 out of the 64 ER–/PgR+/HER2– cases. An endocrine therapy sensitivity score was also calculated according to the subgroups within the ER–/PgR+ group in the 17 cases from cohort 4 (**c**) and in the 64 cases from cohort 2 (**d**). The subgroups within the ER–/PgR+ were evaluated by PAM50 in cohort 4 and by immunohistochemistry in the 64 cases. *P* values of sensitivity score between luminal-like and basal-like are <0.0001 for both sets (Mann–Whitney test). LL, Luminal-like; UN, Undetermined; BL, Basal-like

Survival analysis in 55 out of the 64 ER–/PgR+ cases from FDUSCC according to adjuvant endocrine therapy is shown in Additional file 8: Table S5. Patients with a luminal-like ER–/PgR+ subtype benefited more from sufficient adjuvant endocrine therapy (defined as undergoing cumulative endocrine treatment for more than one year) than insufficient treatment (less than one year or no endocrine therapy; log-rank *P* = 0.06. Fig. 3a). In contrast, the basal-like subgroup did not benefit from endocrine therapy (log-rank *P* = 0.61. Fig. 3b). Because of limited cases and rare events, the survival outcome of multivariate analysis was unavailable.

To find more evidence to support the above findings, we evaluated the sensitivity to endocrine therapy at the molecular level by calculating the ER group score (reflects the endocrine responsiveness) in two sets of ER–/PgR+/HER2– cases. The first set was from cohort 4, including 17 cases, and the second set was from cohort 2, including 64 cases. It seemed that luminal-like cases (identified by either PAM50 classifier or IHC-based TFF1/CK5/EGFR makers) had a higher score of sensitivity to endocrine therapy compared with basal-like cases (Mann–Whitney test *P* values <0.0001 for both sets; Fig. 3c, d). When we combined the two sets using a meta-analytic approach (meta-analysis of continuous outcomes, Hedges’ g method), the luminal-like cases had higher score of sensitivity to endocrine therapy compared with basal-like cases (standardized mean difference of 3.45 with 95 % CI, 2.65–4.26; *P* <0.0001, by a fixed-effects method). Further analysis showed that basal-like ER–/PgR+/HER2– cases had endocrine therapy sensitivity scores as low as those in triple-negative cases (*P* = 0.80, by a fixed-effects method).

## Discussion

In the present study, we systemically investigated the clinicopathologic features and molecular essence of a clinically rare but biologically occurring ER–/PgR+/HER2– phenotype. We revealed that the clinicopathologic features and survival outcomes of this phenotype fell in between ER+/PgR+ and ER–/PgR– and were more similar to the ER–/PgR– phenotype. For the intrinsic subtype of ER–/PgR+ tumors, about 30 % were luminal-like and 60 % were basal-like. Moreover, we developed a feasible IHC-based method using three markers, TFF1, CK5, and EGFR, to determine the prognosis-relevant subtype of each ER–/PgR+ case, which may assist oncologists in making treatment decisions. ER–/PgR+ cases with basal-like characteristics may eliminate long-term but ineffective endocrine therapy and lead to individualized chemotherapy.

In our series, the majority of ER–/PgR+ tumors occurred in younger women with poorly differentiated tumors, which have been observed in triple-negative cases [43]. At the molecular level, about 60 % were associated with a basal-like subtype, while only less than 30 % showed luminal features. Currently, routine clinical evaluation of subtype is most valuable in predicting the response to targeted therapy. Clinical guidelines, such as the St. Gallen consensus, recommend tailoring adjuvant systemic treatment according to subtypes [11]. However, the ER–/PgR+ phenotype is not mentioned in the 2013 St. Gallen consensus, and the recommended treatment is therefore undetermined. Although the ER–/PgR+ phenotype belongs to the “hormone receptor-positive” group and is suggested to use endocrine therapy, its response to endocrine therapy is low [3, 4, 12]. According to a collaborative meta-analysis of individual patient data from 20 trials (n = 21,457) in early breast cancer of about 5 years of tamoxifen versus no adjuvant tamoxifen by Early Breast Cancer Trialists’ Collaborative Group [4], the rate ratios were 0.63 (standard error, 0.03) for ER+/PgR+ disease (*P* <0.00001) but 0.90 (standard error, 0.10) for ER–/PgR+ disease (*P* = 0.35). We herein demonstrated that the majority of ER–/PgR+ cases were actually basal-like, therefore indicating that treatment of ER–/PgR+ cases with long-term endocrine therapy for 5 years or even more is questionable.

Being able to identify the luminal-like subgroup within the ER–/PgR+ phenotype is important. Our study provides, for the first time, an effective and feasible IHC method to distinguish the intrinsic subtype within the ER–/PgR+ phenotype using three markers, TFF1, CK5, and EGFR. TFF1 is an indicator of the functional estrogen-responsive pathway and improves the response to tamoxifen [44]. KRT5 and EGFR are identified as reliable basal markers [25]. Moreover, we identified a significant difference in the sensitivity to endocrine therapy between luminal-like ER–/PgR+ and basal-like ER–/PgR+. Basal-like ER–/PgR+ cases obtained limited benefit from endocrine therapy, while luminal-like ER–/PgR+ cases probably benefited from endocrine therapy despite of ER loss. There are some potential explanations for this. First, in these cases, ER-negativity is falsely negative [5]. Technical failure in ER detection made it difficult to detect positive ER even after re-assessment by IHC. Second, strong evidence exists for the presence of plasma membrane ER (only nuclear staining of ER is recognized as ER-positivity according to the ASCO/CAP guideline [12]). When estrogen binds cell surface ER, membrane-initiated stimulation is able to induce and potentiate the genomic activation of PgR expression [45, 46]. In this situation, endocrine therapy by antagonizing or reducing estrogen may also work.

Our study has some limitations. First, we excluded HER2+ cases and thus our findings could not be applicable in the ER–/PgR+/HER2+ phenotype. Second, although it is better to use the same survival endpoint (BCSS, RFS, or DRFS) for analysis, unfortunately, the various cohorts provide different endpoints and it is impossible to use the same endpoint for analysis. Third, because of limited ER–/PgR+/HER2– cases included in analysis of sensitivity to endocrine therapy and rare survival events, it is still too early to conclude the causal association between basal-like ER–/PgR+ tumors and limited benefit from endocrine therapy. Finally, our study is biased by its retrospective nature. However, due to the very low incidence of the ER–/PgR+/HER2– phenotype, it is impractical to conduct a large-scale prospective trial to test our hypothesis; we therefore must rely on data from the present large retrospective study. Our study uses the data from some prospective observational cohorts and provides a piece of state-of-the-art evidence describing the molecular essence of ER–/PgR+ and how to recognize the subtype of a ER–/PgR+ case using an IHC assay.

## Conclusion

In conclusion, the majority of the ER–/PgR+/HER2– phenotype breast cancer cases are basal-like and a minority is luminal-like. Detecting immunohistochemical TFF1, CK5, and EGFR may help to identify the intrinsic subgroups within this phenotype. Basal-like ER–/PgR+ tumors may obtain limited benefit from endocrine therapy and further large-scale studies will be necessary to validate our findings.

## Additional files

Additional file 1: Figure S1. The study flowchart diagram. FDUSCC, Fudan University Shanghai Cancer Center; NCT, Neoadjuvant chemotherapy; SEER, Surveillance, Epidemiology and End Results program. (PPTX 111 kb) Additional file 2: Table S1. Characteristics of the 64 ER–/PgR+/HER2– phenotype cases from FDUSCC. (DOC 49 kb) Additional file 3: Table S2. Genes and probe sets used to characterize subgroups within ER–/PgR+/HER2– phenotype. (DOC 40 kb) Additional file 4.
**Supplemental Methods.** (DOC 35 kb) Additional file 5: Table S3. PCR Primers of ER group genes and reference genes. (DOC 32 kb) Additional file 6: Table S4. Comparison of characteristics among ER+/PgR+, ER–/PgR+, and ER–/PgR– phenotypes. (DOC 58 kb) Additional file 7: Figure S2. ER and PgR expression in the ER–/PgR+/HER2– phenotype in cohort 4. PP, ER+ and PgR+; NP, ER– and PgR+; NN, ER– and PgR–. (PPTX 79 kb) Additional file 8: Table S5. Survival benefit from adjuvant endocrine therapy in 55 out of the 64 ER–/PgR+/HER2– cases. (DOC 30 kb)

## Abbreviations

ASCO/CAP: American Society of Clinical Oncology/College of American Pathologists
BCSS: Breast cancer-specific survival
CIs: Confidence intervals
CKs: Cytokeratins
DRFS: Distant relapse-free survival
ER: Estrogen receptor
FDUSCC: Fudan University Shanghai Cancer Center
HRs: Hazard ratios
IHC: Immunohistochemistry
PgR: Progesterone receptor
RFS: Relapse-free survival
SEER: Surveillance, Epidemiology, and End Results
