# Supplementary figures and images for: Molecular essence and endocrine responsiveness of estrogen receptor-negative, progesterone receptor-positive, and HER2-negative breast cancer
Source: BMC Med. 2015 Oct 5;13:254. doi: 10.1186/s12916-015-0496-z (PMC4595063; doi:10.1186/s12916-015-0496-z)

## Slide 1
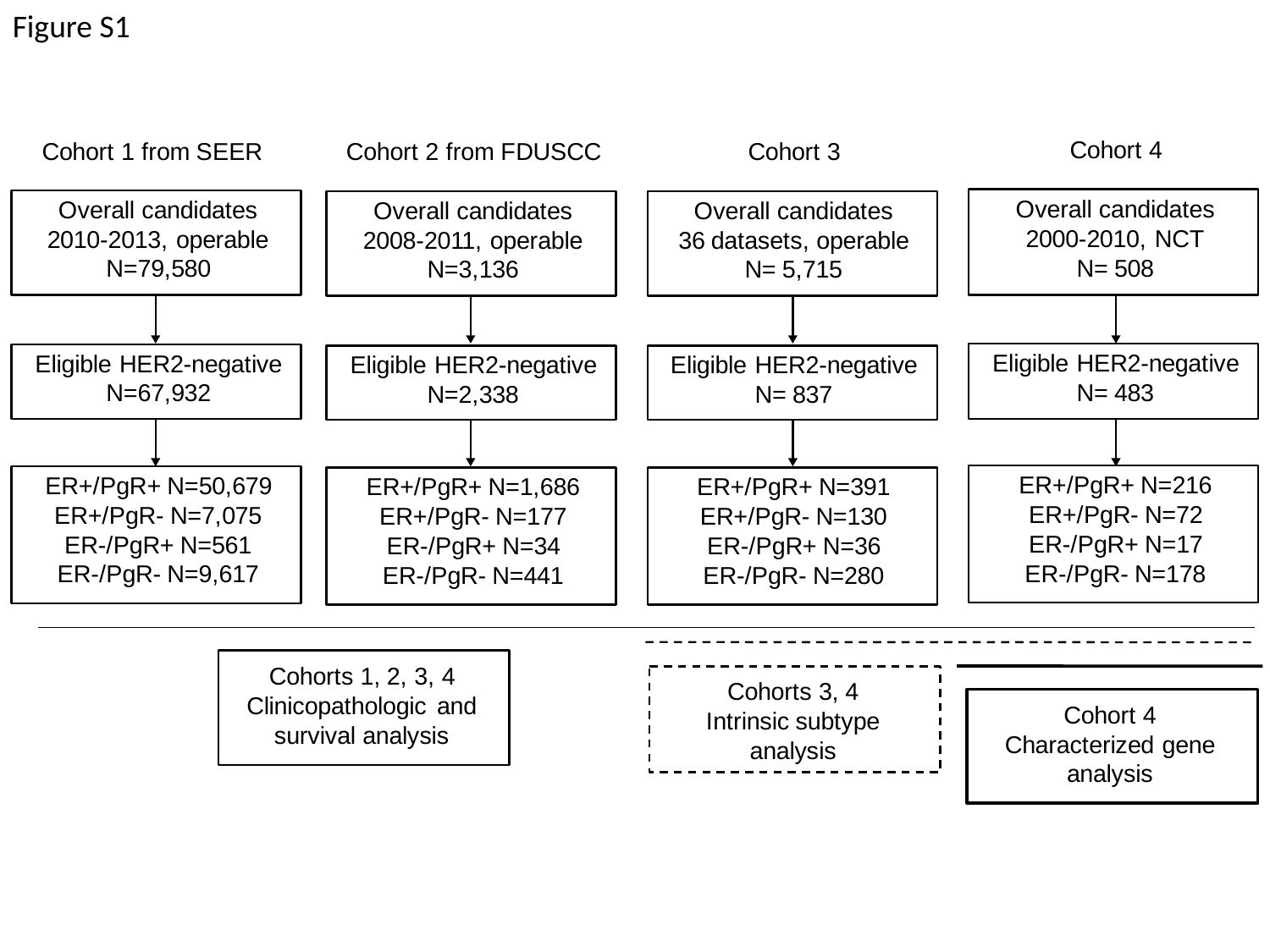

Figure S1

Supplement: Additional file 1: Figure S1. — The study flowchart diagram. FDUSCC, Fudan University Shanghai Cancer Center; NCT, Neoadjuvant chemotherapy; SEER, Surveillance, Epidemiology and End Results program. (PPTX 111 kb) [file 12916_2015_496_MOESM1_ESM.pptx]

## Slide 1
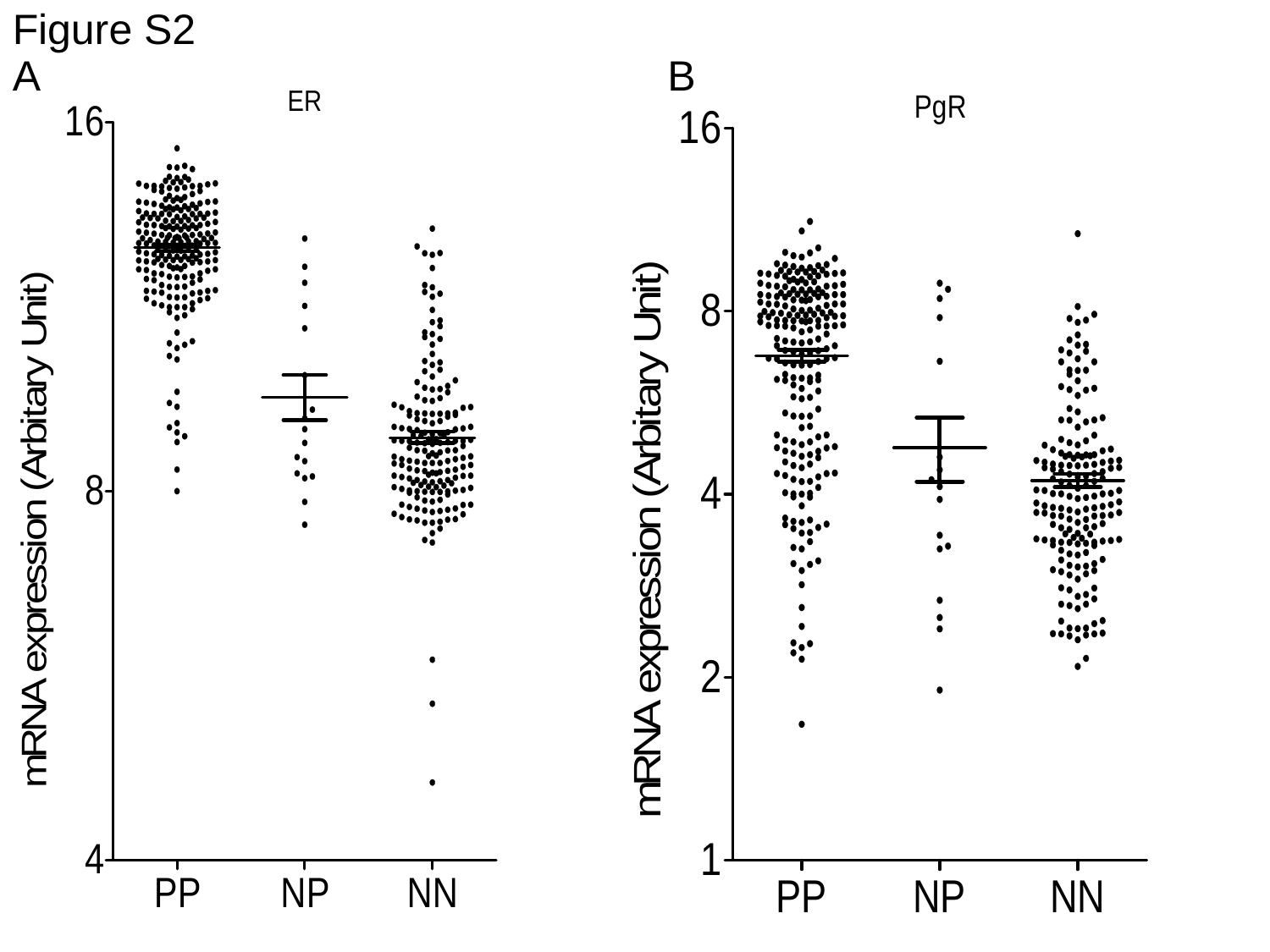

Figure S2
A
B

Supplement: Additional file 7: Figure S2. — ER and PgR expression in the ER–/PgR+/HER2– phenotype in cohort 4. PP, ER+ and PgR+; NP, ER– and PgR+; NN, ER– and PgR–. (PPTX 79 kb) [file 12916_2015_496_MOESM7_ESM.pptx]
