# Supplementary material for: Molecular essence and endocrine responsiveness of estrogen receptor-negative, progesterone receptor-positive, and HER2-negative breast cancer
Source: BMC Med. 2015 Oct 5;13:254. doi: 10.1186/s12916-015-0496-z (PMC4595063; doi:10.1186/s12916-015-0496-z)
Supplement: Additional file 2: Table S1. — Characteristics of the 64 ER–/PgR+/HER2– phenotype cases from FDUSCC. (DOC 49 kb) [file 12916_2015_496_MOESM2_ESM.doc]

Additional file 2: Table S1

Characteristics of the 64 ER-/PgR+/HER2- phenotype cases from FDUSCC

|  | Overall (n=64) | Luminal-like (n=15) | | Uncertain (n=9) | | Basal-like (n=40) | |
| --- | --- | --- | --- | --- | --- | --- | --- |
|  |  | TFF1-positive | | TFF1-negative and CK5/EGFR-negative | | TFF1-negative and CK5/EGFR-positive | |
|  | Number | Number | % | Number | % | Number | % |
| Median age (years) | 52 | 52 |  | 53 |  | 52 |  |
| Size |  |  |  |  |  |  |  |
| T0-1 | 19 | 4 | 26.70% | 4 | 44.40% | 11 | 27.50% |
| T2 | 38 | 7 | 46.70% | 5 | 55.60% | 26 | 65.00% |
| T3-4 | 7 | 4 | 26.70% | 0 | 0.00% | 3 | 7.50% |
| Grade |  |  |  |  |  |  |  |
| I-II | 18 | 5 | 33.30% | 5 | 55.60% | 8 | 20.00% |
| III | 46 | 10 | 66.70% | 4 | 44.40% | 32 | 80.00% |
| Lymph nodes |  |  |  |  |  |  |  |
| Negative | 28 | 8 | 53.30% | 7 | 77.80% | 13 | 32.50% |
| Positive | 36 | 7 | 46.70% | 2 | 22.20% | 27 | 67.50% |
| Adjuvant endocrine therapy |  |  |  |  |  |  |  |
| Insufficient (Never or <1 year) | 22 | 7 | 46.70% | 0 | 0.00% | 15 | 37.50% |
| Sufficient (≥1 years) | 42 | 8 | 53.30% | 9 | 100.00% | 25 | 62.50% |
| Median Follow-up time (months) | 50 | 63 |  | 59 |  | 43 |  |
| Relapse* |  |  |  |  |  |  |  |
| No | 47 | 13 | 86.70% | 7 | 77.80% | 27 | 67.50% |
| Yes | 17 | 2 | 13.30% | 2 | 22.20% | 13 | 32.50% |

FDUSCC, Fudan University Shanghai Cancer Center

* relapse indicates the first event of local, regional, or distant metastasis of breast cancer
