# Supplementary material for: Molecular essence and endocrine responsiveness of estrogen receptor-negative, progesterone receptor-positive, and HER2-negative breast cancer
Source: BMC Med. 2015 Oct 5;13:254. doi: 10.1186/s12916-015-0496-z (PMC4595063; doi:10.1186/s12916-015-0496-z)
Supplement: Additional file 3: Table S2. — Genes and probe sets used to characterize subgroups within ER–/PgR+/HER2– phenotype. (DOC 40 kb) [file 12916_2015_496_MOESM3_ESM.doc]

Additional file 3: Table S2

Genes and probe sets used to characterize subgroups within ER-/PgR+/HER2- phenotype

| Gene | Probe Set Name | EntrezGene | Full Name of Gene |
| --- | --- | --- | --- |
| CDH1 | 201130_s_at | 1012 | CDH13: cadherin 13, H-cadherin (heart) |
| CDH1 | 201131_s_at | 1012 | CDH13: cadherin 13, H-cadherin (heart) |
| CLDN3 | 203953_s_at | 1365 | CLDN3: claudin 3 |
| CLDN3 | 203954_x_at | 1365 | CLDN3: claudin 3 |
| CLDN4 | 201428_at | 1364 | CLDN4: claudin 4 |
| CLDN7 | 202790_at | 1366 | CLDN7: claudin 7 |
| EGFR/HER1 | 201983_s_at | 1956 | EGFR: epidermal growth factor receptor |
| EGFR/HER1 | 201984_s_at | 1956 | EGFR: epidermal growth factor receptor |
| EGFR/HER1 | 210984_x_at | 1956 | EGFR: epidermal growth factor receptor |
| EGFR/HER1 | 211550_at | 1956 | EGFR: epidermal growth factor receptor |
| EGFR/HER1 | 211551_at | 1956 | EGFR: epidermal growth factor receptor |
| EGFR/HER1 | 211607_x_at | 1956 | EGFR: epidermal growth factor receptor |
| ESR1 | 205225_at | 2099 | ESR1: estrogen receptor 1 |
| GREB1 | 205862_at | 9687 | GREB1: growth regulation by estrogen in breast cancer 1 |
| GREB1 | 210562_at | 9687 | GREB1: growth regulation by estrogen in breast cancer 1 |
| GREB1 | 210855_at | 9687 | GREB1: growth regulation by estrogen in breast cancer 1 |
| KRT14 | 209351_at | 3861 | KRT14: keratin 14 |
| KRT17 | 205157_s_at | 3872 | KRT17: keratin 17 |
| KRT17 | 212236_x_at | 3872 | KRT17: keratin 17 |
| KRT5 | 201820_at | 3852 | KRT5: keratin 5 |
| PDZK1 | 205380_at | 5174 | PDZK1: PDZ domain containing 1 |
| PGR | 208305_at | 5241 | PGR: progesterone receptor |
| TFF1/pS2 | 205009_at | 7031 | TFF1: trefoil factor 1 |
