# Supplementary material for: Molecular essence and endocrine responsiveness of estrogen receptor-negative, progesterone receptor-positive, and HER2-negative breast cancer
Source: BMC Med. 2015 Oct 5;13:254. doi: 10.1186/s12916-015-0496-z (PMC4595063; doi:10.1186/s12916-015-0496-z)
Supplement: Additional file 4. — Supplemental Methods. (DOC 35 kb) [file 12916_2015_496_MOESM4_ESM.doc]

**Supplemental Methods**

**Formalin-fixed, paraffin-embedded (FFPE) Sample Collection and RNA Extraction**

FFPE sample collection and RNA extraction was performed as previously described. Briefly, selected blocks with cancer cells taking up at least 80% of the section area were used. All sample sections were stained with H&E and were examined by two independent pathologists. After paraffin removed from samples by using xylene followed by absolute ethanol washes, tissues on microscope slides were scratched from slides. The RNA was extracted by using High Pure Paraffin RNA Isolation Kit (Roche, Mannheim, Germany) and Recover All Total Nucleic Acid Isolation kit (Ambion, Austin, TX, USA) according to the manufacturer’s protocols.

**Gene Expression Analysis by Quantitative Reverse Transcription Polymerase Chain Reaction (QRT-PCR)**

Reverse transcription (RT) was performed using the High-capacity cDNA Reverse Transcription Kit (Applied Biosystem, Foster City, CA, USA) with random primers. The cDNA was subjected to quantitative PCR with the SYBR Green fluorescent-based assay (TaKaRa, Japan) as previously described in a fluorescence temperature cycler (Opticon, MJ Research), using the standard curves method. All samples were run in triplicate. PCR primers are listed in **Supplemental Table S3** as previously described and two genes, ACTB and GAPDH, were measured for normalization.

**References:**

1. Sun B, Zhang F, Wu SK, Guo X, Zhang LL, Jiang ZF, Wang DM, Song ST: **Gene expression profiling for breast cancer prognosis in Chinese populations**. *Breast J* 2011, **17**(2):172-179.

2. Aldea C, Alvarez CP, Folgueira L, Delgado R, Otero JR: **Rapid detection of herpes simplex virus DNA in genital ulcers by real-time PCR using SYBR green I dye as the detection signal**. *J Clin Microbiol* 2002, **40**(3):1060-1062.
