# Supplementary material for: Molecular essence and endocrine responsiveness of estrogen receptor-negative, progesterone receptor-positive, and HER2-negative breast cancer
Source: BMC Med. 2015 Oct 5;13:254. doi: 10.1186/s12916-015-0496-z (PMC4595063; doi:10.1186/s12916-015-0496-z)
Supplement: Additional file 5: Table S3. — PCR Primers of ER group genes and reference genes. (DOC 32 kb) [file 12916_2015_496_MOESM5_ESM.doc]

Additional file 5: Table S3

PCR Primers of ER group genes and reference genes

| **Gene name** |  | **Primer sequence (5′-3′)** |
| --- | --- | --- |
| ACTB | ACTBF | AGCAAGCAGGAGTATGACG |
|  | ACTBR | GAAAGGGTGTAACGCAACT |
| GAPDH | GAPDHF | ACAGTCAGCCGCATCTTCTT |
|  | GAPDHR | ACGACCAAATCCGTTGACTC |
| BCL2 | BCL2F | AAGCCCCAAAAGGAGAAGAA |
|  | BCL2R | GTCATTCTGGCCTCTCTTGC |
| PGR | PGRF | GCATCAGGCTGTCATTATGG |
|  | PGRR | AGTAGTTGTGCTGCCCTTCC |
| SCUBE2 | SCUBE2F | TGACAATCAGCACACCTGCAT |
|  | SCUBE2R | GCCTCCTTGCAGATGTGACT |
| ESR | ESRF | TCCAGCACCCTGAAGTCTCT |
|  | ESRR | GCCATCAGGTGGATCAAAGT |

F, forward primer; R, reverse primer.
