# Supplementary material for: Molecular essence and endocrine responsiveness of estrogen receptor-negative, progesterone receptor-positive, and HER2-negative breast cancer
Source: BMC Med. 2015 Oct 5;13:254. doi: 10.1186/s12916-015-0496-z (PMC4595063; doi:10.1186/s12916-015-0496-z)
Supplement: Additional file 6: Table S4. — Comparison of characteristics among ER+/PgR+, ER–/PgR+, and ER–/PgR– phenotypes. (DOC 58 kb) [file 12916_2015_496_MOESM6_ESM.doc]

Additional file 6: Table S4

Comparison of characteristics among ER+/PgR+, ER-/PgR+, and ER-/PgR- phenotypes

|  | Cohort 1: SEER (n=60857) | | | | | | | Cohort 2: FDUSCC (n=2161) | | | | | | | Cohort 3: Publicly available cases (n=707) | | | | | | | Cohort 4: Publicly available NCT cases (n=411) | | | | | | |
| --- | --- | --- | --- | --- | --- | --- | --- | --- | --- | --- | --- | --- | --- | --- | --- | --- | --- | --- | --- | --- | --- | --- | --- | --- | --- | --- | --- | --- |
|  | ER+/PgR+ | % | ER-/PgR+ | % | ER-/PgR- | % | P | ER+/PgR+ | % | ER-/PgR+ | % | ER-/PgR- | % | P | ER+/PgR+ | % | ER-/PgR+ | % | ER-/PgR- | % | P | ER+/PgR+ | % | ER-/PgR+ | % | ER-/PgR- | % | P |
| Age (median), years | 61 |  | 55 |  | 57 |  | <0.001 | 55 |  | 53 |  | 54 |  | 0.02 | 55 |  | 51 |  | 53 |  | 0.005 | 49 |  | 47 |  | 49 |  | 0.94 |
| Size |  |  |  |  |  |  | <0.001 |  |  |  |  |  |  | <0.001 |  |  |  |  |  |  | 0.20 |  |  |  |  |  |  | 0.038 |
| T0-1 | 33732 | 66.6 | 249 | 44.4 | 4255 | 44.2 |  | 872 | 51.7 | 10 | 29.4 | 169 | 38.3 |  | 71 | 37.6 | 15 | 60.0 | 46 | 38.0 |  | 17 | 7.9 | 3 | 17.6 | 9 | 5.1 |  |
| T2 | 13654 | 26.9 | 247 | 44.0 | 4045 | 42.1 |  | 684 | 40.6 | 20 | 58.8 | 224 | 50.8 |  | 101 | 53.4 | 10 | 40.0 | 65 | 53.7 |  | 108 | 50.0 | 12 | 70.6 | 85 | 47.8 |  |
| T3-4 | 3293 | 6.5 | 65 | 11.6 | 1317 | 13.7 |  | 130 | 7.7 | 4 | 11.8 | 48 | 10.9 |  | 17 | 9.0 | 0 | .0 | 10 | 8.3 |  | 91 | 42.1 | 2 | 11.8 | 84 | 47.2 |  |
| Lymph nodes |  |  |  |  |  |  | <0.001 |  |  |  |  |  |  | 0.28 |  |  |  |  |  |  | 0.019 |  |  |  |  |  |  | 0.051 |
| Negative | 35683 | 70.4 | 359 | 64.0 | 6264 | 65.1 |  | 824 | 49.0 | 15 | 44.1 | 232 | 53.0 |  | 120 | 43.6 | 22 | 66.7 | 97 | 52.2 |  | 78 | 36.1 | 5 | 29.4 | 44 | 24.7 |  |
| Positive | 14996 | 29.6 | 202 | 36.0 | 3353 | 34.9 |  | 856 | 51.0 | 19 | 55.9 | 206 | 47.0 |  | 155 | 56.4 | 11 | 33.3 | 89 | 47.8 |  | 138 | 63.9 | 12 | 70.6 | 134 | 75.3 |  |
| Grade |  |  |  |  |  |  | <0.001 |  |  |  |  |  |  | <0.001 |  |  |  |  |  |  | <0.001 |  |  |  |  |  |  | <0.001 |
| I | 15535 | 31.7 | 13 | 2.4 | 204 | 2.2 |  | 36 | 2.1 | 0 | .0 | 1 | .2 |  | 82 | 22.5 | 1 | 2.9 | 1 | .4 |  | 24 | 11.8 | 1 | 5.9 | 1 | .6 |  |
| II | 24905 | 50.8 | 100 | 18.5 | 1579 | 17.0 |  | 1321 | 78.4 | 10 | 29.4 | 155 | 35.1 |  | 161 | 44.2 | 9 | 26.5 | 27 | 10.5 |  | 112 | 54.9 | 4 | 23.5 | 22 | 13.5 |  |
| III and UD | 8626 | 17.6 | 428 | 79.1 | 7488 | 80.8 |  | 329 | 19.5 | 24 | 70.6 | 285 | 64.6 |  | 121 | 33.2 | 24 | 70.6 | 229 | 89.1 |  | 68 | 33.3 | 12 | 70.6 | 140 | 85.9 |  |

FDUSCC, Fudan University Shanghai Cancer Center; NCT, neoadjuvant chemotherapy; SEER, Surveillance, Epidemiology and End Results program; UD, undifferentiated.
