# Supplementary material for: Molecular essence and endocrine responsiveness of estrogen receptor-negative, progesterone receptor-positive, and HER2-negative breast cancer
Source: BMC Med. 2015 Oct 5;13:254. doi: 10.1186/s12916-015-0496-z (PMC4595063; doi:10.1186/s12916-015-0496-z)
Supplement: Additional file 8: Table S5. — Survival benefit from adjuvant endocrine therapy in 55 out of the 64 ER–/PgR+/HER2– cases. (DOC 30 kb) [file 12916_2015_496_MOESM8_ESM.doc]

Additional file 8: Table S5

Survival benefit from adjuvant endocrine therapy in 55 out of the 64 ER-/PgR+/HER2- cases

|  | Insufficient Adjuvant Endocrine Therapy | | Sufficient Adjuvant Endocrine Therapy | | Univariate log-rank P |
| --- | --- | --- | --- | --- | --- |
|  | No relapse | Relapse | No relapse | Relapse |
| Overall group | 14 | 8 | 36 | 7 | 0.33 |
| Luminal-like | 5 | 2 | 8 | 0 | 0.06 |
| Basal-like | 9 | 6 | 18 | 7 | 0.61 |

Luminal-like group and basal-like group defined by integrated expression of TFF1, EGFR, and CK5.
